# Prevalence and associated risk factors of peripheral artery disease in virologically suppressed HIV-infected individuals on antiretroviral therapy in Kwara state, Nigeria: a cross sectional study

**DOI:** 10.1186/s12889-019-7496-4

**Published:** 2019-08-20

**Authors:** Chidozie Elochukwu Agu, Ikenna Kingsley Uchendu, Augusta Chinyere Nsonwu, Chukwugozie Nwachukwu Okwuosa, Peter Uwadiegwu Achukwu

**Affiliations:** 1 Prime Health Response Initiative (PHRI)-sub-recipients of Global Fund HIV Impact Project, Ilorin, Kwara State Nigeria; 2 Department of Medical Laboratory Science, Faculty of Health Science and Technology, College of Medicine, University of Nigeria Enugu Campus, Enugu, Nigeria; 3 Department of Medical Laboratory Science, Faculty of Allied Medical Science, University of Calabar, Calabar, Cross River State Nigeria

**Keywords:** HIV, Antiretroviral therapy, Cardiovascular disease, Metabolic syndrome, Dyslipidemia

## Abstract

**Background:**

The association between HIV and cardiovascular disease (CVD) has been reported in several studies. However, there is paucity of information on the prevalence of subclinical disease as well as its associated risk factors in sub-Saharan African population. The aim of this study was to determine the prevalence and associated risk factors of peripheral artery disease (PAD) among virologically suppressed HIV-infected participants in Kwara State, Nigeria.

**Methods:**

This study was conducted between July 2018 and December 2018. A total of 150 HIV-infected participants aged between 20 and 55 years and 50 HIV non-infected age-matched controls were randomly recruited in the study. Sociodemographic, anthropometric and clinical data were collected using a well-structured questionnaire. Ankle brachial index (ABI) was measured, PAD was defined as ABI of < 0.9. Cryopreserved serum was used to evaluate lipid profile parameters. Student’s t-test and Chi-square were used to compare continuous and categorical variables. Associations of CVD risk factors and clinical data, and lipid profile with low ABI were assessed using logistic regression analysis.

**Results:**

The study participants had a mean age of 43.73 ± 8.74, majority were females (72.7%) with a mean duration on ART of 7.73 ± 3.52 years. Hypertension was present in 15.9%, diabetes 4%, family history of CVD 8.6% and metabolic syndrome 17.3% in the study group. The study participants recorded significantly lower mean values for ABI, HDL-C and significantly higher mean values of TG (*P* < 0.05) compared to the control group. The prevalence of low ABI (14.6%) was higher in the study group compared to the control group (2%). A significantly negative correlation between ABI and duration on ART (*r* = − 0.163, *P* = 0.041) and a positive correlation between viral load and TG were observed in the study group. TC (OR 1.784, *P* = 0.011), LDL-C (OR 1.824, *P* = 0.010) and CD4 cell count < 200 cells/mm^3^ (OR 2.635, *P* = 0.364) were associated with low ABI in the participants.

**Conclusion:**

Viral suppression with combined antiretroviral therapy and long term treatment is associated with dyslipidaemia, with increased risk of PAD. Prevalence of PAD in virologically-suppressed individuals does not differ from the controls in the population studied.

**Electronic supplementary material:**

The online version of this article (10.1186/s12889-019-7496-4) contains supplementary material, which is available to authorized users.

## Background

In developed countries, there is significant improvement in the prognosis of People Living with HIV (PLHIV), who receive antiretroviral treatment (ART); as a result of the availability of well-established access to care and ART [1, 2]. In the last 20 years, there has been consistent decline in the incidence and mortality of AIDS-defining illnesses linked to severe end-stage immune suppression, whereas the role of non-AIDS comorbidities has risen [3–6]. These reports have come mainly from high- and middle-income countries [7, 8], with very few reports from low-income countries, particularly sub-Saharan Africa [9, 10]. Although reports from sub-Saharan Africa indicated that tuberculosis, sepsis, advanced HIV disease and pulmonary infections are the leading causes of death [11]. Furthermore, CVD (cardiovascular disease) is now increasingly implicated as the cause of death among HIV-infected individuals in sub-Saharan Africa.

Greater prevalence and risk of CVD has been reported in long-term–treated patients than age-matched uninfected adults, without sufficient knowledge for the reason [7], and the underlying mechanism causing the high risk of non-AIDS complications is most likely multifactorial and includes comorbid conditions and toxicity from antiretroviral therapy [12, 13].

Although the relative risk of CVD is increased in patients with HIV, one limitation in studying CVD in this generally young patient population has been the relatively low absolute event rate in terms of CVD deaths [14]. Peripheral artery disease **(**PAD) is highly associated with atherosclerosis and is a predictor of cardiovascular outcomes [14–17]. Ankle brachial index (ABI) has been used to document the burden of subclinical CVD, and better understand the underlying pathophysiological mechanisms, and follow response to treatment interventions. Ankle-brachial index (ABI) is the screening tool for PAD. An ABI ≤ 0.9 (low ABI) is diagnostic of PAD [18]. The ABI between 0.91–0.99 (borderline values) is significantly considered prognostic in higher cardiovascular mortality. The ABI ≥ 1.3 (high ABI) suggests rigidity and non-compressibility of lower limb arteries. Abnormal ABI value in asymptomatic patients is associated with a higher incidence of CVD [19].

This study was aimed to evaluate the prevalence and associated risk factors of peripheral artery disease (PAD) in virologically suppressed HIV-infected participants in Ilorin, Kwara State, Nigeria.

## Methods

### Study area

The study was conducted in Kwara State, a State in western Nigeria, located within the North central geopolitical zone, commonly referred to as the Middle belt with a population of 2,365,353 [20]. The primary ethnic group is Yoruba, with significant Nupe, Bariba and Fulani minorities. Kwara State consists of sixteen (16) Local Government Areas. They are: Asa, Baruten, Edu, Ekiti, Ifelodun, Ilorin East, Ilorin South, Ilorin West, Irepodun, Isin, Kaiama, Moro, Offa, OkeEro, Oyun and Pategi.

### Study design

A cross sectional study design was adopted for this study. Informed consents were obtained from all participants before being enrolled into the study. Socio-demographic and behavioral data were collected using a well-structured questionnaire after which blood samples were drawn from the participants. The study partic.ipants include HIV-positive male and females aged between 20 and 50 years who were attending HIV clinic in University of Ilorin Teaching Hospital (UITH) and Sabo Oke medical center, Ilorin, Kwara state for routine checkup, and antiretroviral therapy (ART) pick up and control group of un-infected health participants resident within the same region. The study was conducted in six (6) months between July 2018 and December 2018.

### Sample size determination

The sample size was calculated using Fischer’s formula

Sample size $$ \left(\mathrm{n}\right)=\frac{{\mathrm{Z}}^2\mathrm{P}\ \left(1\hbox{-} \mathrm{P}\right)}{{\mathrm{D}}^2} $$

Using a prevalence of 11% (prevalence of cardiovascular disease among people living with HIV from the Data Collection on Adverse Events of Anti-HIV Drugs (D:A:D study) [21].

Z = statistics for the level of 95% confidence interval (1.96)

P = prevalence

D = Desired degree of accuracy; here taken to be 0.05

Sample size $$ \left(\mathrm{n}\right)=\frac{(1.96)^2\mathrm{P}\ \left(1\hbox{-} \mathrm{P}\right)}{{\mathrm{D}}^2} $$

$$ \mathrm{n}=\frac{3.84\times 0.11\ \left(1-0.11\right)}{0.05\ \mathrm{x}\ 0.05} $$

$$ \mathrm{n}=\frac{3.84\times 0.11\ (0.89)}{0.0025} $$

$$ \mathrm{n}=150 $$

Thus, a total of 150 HIV- infected participants who have been on antiretroviral therapy (ART) for 6 months and above were recruited into the study.

### Subject selection

A total of 200 participants, made up of 150 HIV-infected participants and 50 un-infected control groups within the age range of 20–50 years, were randomly recruited in the study. This study was conducted between July 2018 and December 2018. The study group participants were recruited from Sabo Oke Medical Center Ilorin; a Global Fund supported site and the ART treatment and care unit, University of Ilorin Teaching Hospital (UITH) supported by PEPFAR (President’s Emergency Plan for AIDS Relief). Medical history along with physical examination, including height, weight, waist and hip measurement, brachial and ankle blood pressure were obtained for all the participants enrolled in the study.

### Inclusion criteria

HIV-infected adults presently on stable ART for at least 6 months without a known coronary heart disease (CHD) or diabetes mellitus and with a viral RNA < 1000 copies/ml, between the ages of 18–50 years.

### Exclusion criteria

HIV-infected adults who have defaulted from ART use, with a known coronary heart disease or diabetes mellitus, those who have been on statin for at least 6 months, those with known co-infections such as tuberculosis, hepatitis B and C, with a viral RNA > 1000 copies/ml or those with advanced symptoms of the disease i.e. AIDS.

### Data collection tools

The questionnaire used in this study was specifically developed for this study (Additional file 1). A detailed questionnaire interview was conducted to collect personal data on each participant enrolled in the study, detailing his/her demographic characteristics, family history of cardiovascular diseases was based on the National Cholesterol Education Program Adult Treatment Panel III (NCEP ATP III) criteria, past and current medication usage, presence of co-infections and HIV disease characteristics (duration of HIV infection, duration of ART treatment, viral RNA count, nadir and current CD4+ T-cell count, and past and current ART regimen) [22].

### Ankle brachial index (ABI) measurement

The ABI was determined using a hand-held continuous wave doppler device 8 MHz probe, as described by Kwiatkowska et al. [22] and Olalla et al. [23]. Briefly, the examination was done after 5 min of rest in the supine position. A pressure cuff was used, placed on either arm and then above the ankles. The probe was placed at the cubital fossa, medial malleolus and dorsal foot for systolic blood pressure detection at the brachial artery, left posterior tibial and dorsalis pedis artery respectively. The cuff was inflated to 20 mm above the audible pulse signal and then slowly emptied until the first pulse signal was detected. Ankle brachial index value was determined by taking the higher pressure of the two arteries at the ankles divided by the highest brachial systolic blood pressures. Using the formula below, ABI was calculated as:

$$ \mathrm{ABI}=\frac{\mathrm{Highest}\ \mathrm{systolic}\ \mathrm{blood}\ \mathrm{pressure}\ \mathrm{in}\ \mathrm{the}\ \mathrm{Ankles}}{\mathrm{Highest}\ \mathrm{systolic}\ \mathrm{blood}\ \mathrm{pressure}\ \mathrm{in}\ \mathrm{both}\ \mathrm{arms}} $$

Interpretation of ankle brachial index measurements: Normal ABI = 1.0–1.4

Borderline ABI = 0.91–0.99,

PAD = < 0.9

### Anthropometric measurements

Anthropometric parameters such as height (m), weight (kg), waist circumference (cm), hip circumference (cm) were determined as described by Schienkiewitz et al. [24]. Briefly, weight and height were measured with the subjects wearing light clothing and without shoes.Weight was estimated in kilogram using a balanced scale; height was measured in meters using a wall-mounted ruler with the participant standing with feet together and with head, shoulder, buttocks and heels touching the wall. Waist and hip circumference were estimated to the nearest 0.1 cm using a flexible but inelastic measuring tape; waist circumference was taken between the costal margin and the iliac crest in the mid-auxillary line around the gluteal region [24, 25]. Body mass index was calculated as weight in kilogram divided by the square of the height in metres (kg/m^2^), waist-to-hip (WHR) was calculated by dividing the measurement of the waist (cm) by that of the hip (cm) and was used together with waist circumference as an index of central obesity.

The following definitions were used, general obesity a BMI of ≥30 kg/m^2^, central obesity – a waist circumference of ≥80 cm in women or ≥ 94 cm in men, waist-to-hip ratio of ≥0.90 in men or ≥ 0.85 in women. Normal weight, as BMI of 18.5–24.9 kg/m^2^ [25].

### Definition of cardiovascular disease (CVD) risk factors

#### Dyslipidaemia

Dyslipidaemia was defined as either elevated TG levels ≥1.7 mmol/l (150 mg/dl), reduced HDL-cholesterol < 0.9 mmol/l (40 mg/dl) formen and < 1.29 mmol/l (50 mg/dl) in women, or specific treatment for previously identified hypertriglyceridaemia and/or reduced HDL-cholesterol (NCEP criteria).

#### Elevated blood pressure/hypertension

Elevated blood pressure was defined as systolic pressure ≥ 130 mmHg, diastolic ≥85 mmHg, or antihypertensive therapy for initially detected hypertension [26].

#### Hyperglycaemia

Hyperglycaemia was defined as the presence of either fasting plasma glucose of (≥ 5.6 mmol/l or 100 mg/dl) or previously detected diabetes [27].

#### Metabolic syndrome (MetS)

Participants defined as having the MetS, had central obesity (defined as waist circumference) ≥ 94 cm for males and ≥ 80 cm for females in addition with any two of four factors. These four factors are:

- Elevated triglyceride level: ≥ 1.7 mmol/l (150 mg/dl) • decreased HDL-cholesterol: < 1.03 mmol/l (40 mg/dl) in males and < 1.29 mmol/l (50 mg/dl) in females (or specific treatment for these abnormal lipid values).
- Elevated blood pressure (systolic BP ≥ 130 or diastolic BP ≥ 85 mmHg) (or treatment of previously detected hypertension).
- Elevated fasting plasma glucose [FPG ≥ 5.6 mmol/l (100 mg/dl)] (or previously detected diabetes) [27].

#### Smoking

Participants were defined as smokers if they reported current cigarette smoking or had ceased from smoking within the last 2 years.

### Sample collection

Ten milliliters (10mls) of blood was collected from the ante-cubital vein by venipuncture after at least a 12-h overnight fast. Four milliliters (4mls) was dispensed into EDTA container spun at 5000r.p.m. for 5 min, from which 2mls of plasma was transferred into fluoride oxalate container for estimation of fasting plasma glucose. The remaining 6mlswas transferred into a plain container, allowed to clot at room temperature, spun at 5000r.p.m. for 5 min; the serum was separated and cryopreserved for determination of fasting lipid profile.

### Biochemical analysis

#### Measurement of lipid profile parameters

Total cholesterol (TC) was estimated using cholesterol oxidase method as described by Fredrickson et al. [28]. HDL-C was determined using Precipitation method as described by Albers et al. [29]. Triglyceride (TG) was estimated using glycerolphosphate oxidase method as described by Fossati and Prencipe [30]. The LDL-C cholesterol concentration was calculated from the total cholesterol concentration, HDL-cholesterol concentration and the triglyceride concentration using the Friedewald formula [31]:

$$ \mathrm{LDL}-\mathrm{C}\ \left(\mathrm{mmol}/\mathrm{L}\right)=\mathrm{Total}\ \mathrm{cholesterol}-\mathrm{HDL}-\mathrm{C}-\mathrm{TG}/2.2 $$

#### Determination of fasting plasma glucose

Fasting blood glucose was determined using glucose oxidase method. The test kit was obtained from Randox International, UK.

#### Statistical analysis

The data generated were analyzed using Microsoft Excel, Statistical Package for Social Science (SPSS version 20.0, California Inc.) and GraphPad prism (GraphPad Software, Inc.). Data was expressed as mean ± standard deviation, data among groups was compared using a one-way analysis of variance (ANOVA), and post hoc analysis with Tukey’s test. Correlation analysis was done using Pearson’s correlation and was used to estimate inter-variable association between various selected parameters. Values of (*p* < 0.05) were considered statistically significant.

## Results

### Participant characteristics

Ankle brachial index (ABI), anthropometric measurements, lipid profile parameters and fasting plasma glucose (FPG) were evaluated in a total of 200 participants; 150 virologically suppressed (VL < 1000 copies/ml, WHO standard for Low- and middle-income countries) HIV-infected participants on antiretroviral therapy (ART) and 50 uninfected control group. Majority of the study participants were females 109 (72.7%) while 41 (27.3%) were males with a mean age of 43.73 ± 8.74 years. Based on the clinical parameters evaluated, the study participants recorded a mean systolic blood pressure (SBP) of 122 ± 21, diastolic blood pressure (DBP) of 81 ± 8, had a mean 7.73 ± 3.52 years duration of HIV infection, 7.47 ± 3.42 years duration on antiretroviral therapy, mean nadir/baseline CD4+ T-cell count of 263.18 ± 229.58 cells/mm^3^, mean current CD4+ T-cell count of 505.42 ± 265.85 cells/mm^3^ and mean HIV viral RNA count of 40.95 ± 111.61 copies/ml. With regards to drug regimen, 93 (61.6%) of the study participants were on the first line fixed dose regime; zidovudine/lamivudine/nevirapine (AZT/3TC/NVP), 46 (30.5%) were on tenofovir/lamivudine/efavirenz (TDF/3TC/EFV) while 11 (7.3%) were on protease inhibitor; lopinavir/ritonavir (LPV/r) (Table 1).

**Table 1:** Demographics, anthropometric parameters and clinical characteristics of virologically suppressed HIV-infected and un-infected control participants in the study

Participants (n)	Study group (*n* = 150)	Control group (*n* = 50)	*P*-value
Age (yrs.)	43.73 ± 8.74	43.47 ± 8.87	0.714
Gender			
Male (%)	41 (27.3)	23 (46)	0.091
Female (%)	109 (72.7)	27 (54)	0.084
Clinical characteristics			
ABI	0.92 ± 0.08	0.94 ± 0.05	0.175
SBP (mmHg)	121 ± 21	116 ± 17	0.110
DBP (mmHg)	81 ± 8	76 ± 6	0.001*
BMI (kg/m^2^)	25.43 ± 5.46	25.67 ± 4.93	0.778
BMI 18.5–24. kg/m^2^ (%)	78 (52)	26 (52)	0.380
BMI 25–29.9 kg/m^2^ (%)	38 (25.3)	13 (26)	0.250
BMI ≥ 30 kg/m^2^ (%)	34 (22.7)	11 (22)	0.350
WC (cm)	90.13 ± 11.52	90.58 ± 11.13	0.808
WHR	0.88 ± 0.048	0.88 ± 0.053	0.486
Duration of HIV infection (yrs.)	7.73 ± 3.52	N/A	
Duration on ART (yrs.)	7.47 ± 3.42	N/A	
Nadir CD4^+^ T cell count (cells/mm^3^)	263 ± 230	N/A	
Current CD4^+^ T cell count (cells/mm^3)^	505 ± 266	N/A	
HIV viral RNA count (copies/ml)	40.95 ± 111.61	N/A	
Undetectable viremia (< 20 copies/ml)	104 (69.3)		
Detectable viremia (≥20 copies/ml)	46 (30.7)	N/A	
ART Drug regime			
NRTI/NNRTI (AZT/3TC/NVP) (%)	93 (62.0)	N/A	
NRTI/NNRTI (TDF/3TC/EFV) (%)	46 (30.7)	N/A	
NRTI/Protease inhibitor (TDF/3TC/LPV/r) (%)	11 (7.3)	N/A	

Data are presented as mean ± standard deviation or numbers (percentages). * = significant at *P* < 0.05. *PAD* Peripheral artery disease, *ABI* Ankle brachial index, *SBP* Systolic blood pressure, *DBP* Diastolic blood pressure, *BMI* Body mass index, *WC* Waist circumference, *WHR* Waist to hip ratio, *CVD* Cardiovascular disease, *NRTI* Nucleoside reverse transcriptase inhibitor; *NNRTI* Non-nucleoside reverse transcriptase inhibitor, *AZT/3TC/NVP* Zidovudine/lamivudine/nevirapine, *TDF/3TC/EFV* Tenofovir/lamivudine/efavirenz, *LPV/r* Lopinavir/ritonavir, *N/A* Not applicable

### Comparison of ankle brachial index measurement and cardiovascular risk factors in the study and control group

Triglycerides and fasting plasma glucose were significantly higher in the study group compared to the control group while the control group recorded significantly higher HDL-C compared to the study group (*P* < 0.05). Based on ankle brachial index category, the study participants recorded a higher prevalence of low ABI; 22 (14.6%) compared to the control group 3 (2%), normal ABI of 42 (27.8%) compared to 16 (10.6%) recorded by the control group and borderline ABI of 86 (57%) compared to 31 (20.5) recorded by the control group. The study group also recorded a higher prevalence rate of metabolic syndrome; 26 (17.3%) and diabetes mellites 6 (4%) compared to 5 (10%) for metabolic syndrome and 1 (2%) for diabetes mellitus recorded by the controls. The control group however recorded higher prevalence of family history of CVD 20 (13.2%) compared to the study 13 (8.6%) (Table 2).

**Table 2:** Comparison of ankle brachial index and cardiovascular risk factorsbetween the study and control group using student’s t-test and chi-square

Parameter	Study group (*n* = 150)	Control group (*n* = 50)	*P*-value
ABI	0.92 ± 0.08	0.94 ± 0.05	0.175
Normal 1.0–1.3 (%)	42 (27.8)	16 (10.6)	0.589
Borderline 0.9–0.99 (%)	86 (57.0)	31 (20.5)	0.562
PAD < 0.90 (%)	22 (14.6)	3 (2.0)	0.108
TC (mmol/L)	4.65 ± 0.68	4.61 ± 0.98	0.453
TG (mmol/L)	2.27 ± 0.21	2.20 ± 0.14	0.001*
HDL-C (mmol/L)	1.12 ± 0.08	1.19 ± 0.07	0.001*
LDL-C (mmol/L)	2.51 ± 0.70	2.42 ± 0.94	0.236
FPG (mmol/L)	4.51 ± 0.80	4.14 ± 0.68	0.001*
Family history of CVD (%)	13 (8.6)	20 (13.2)	0.355
Diabetes (%)	6 (4.0)	1 (2.0)	0.505
Hypertension (%)	24 (15.9)	5 (3.3)	0.297
Metabolic syndrome (%)	26 (17.3)	5 (10.0)	0.215

Data are presented as mean ± standard deviation or numbers (percentages). * = significant at *P* < 0.05. *PAD* Peripheral artery disease, *ABI* Ankle brachial index, *SBP* Systolic blood pressure, *DBP* Diastolic blood pressure, *BMI* Body mass index, *WC* Waist circumference, *WHR* Waist to hip ratio, *CVD* Cardiovascular disease, *TC* Total cholesterol, *TG* triglyceride, *HDL-C* High density lipoprotein cholesterol, *LDL-C* Low density lipoprotein cholesterol, *FPG* Fasting plasma glucose

### Effect of duration on ART and drug regime class on ABI, CD4^+^ T cell count and cardiovascular risk factors in the study group

Based on duration of ART, age, ankle brachial index and current CD4+ T cell count significantly varied among the groups (*p* < 0.05). Study participants on ART for ≥10 years recorded significantly lower ankle brachial index and higher age compared to participants on drugs for 1–5 and 6–9 years respectively (Table 3). Based on ART regime, waist -hip ratio, ABI, current CD4+ T cell count and FPG varied significantly among the three ART drug regime group. ABI and FPG were significantly higher in study participants on TDF/3TC/EFV regime compared to those on AZT/3TC/NVP. WHR was significantly higher in study participants on AZT/3TC/NVP compared to those on LPV/r (Table 4).

**Table 3:** Comparison of cardiovascular risk markers and clinical characteristics based on duration on antiretroviral therapy (ART) in HIV-infected study participants using one-way ANOVA

Parameter	≤5 years (*n* = 49)	6–9 years (*n* = 50)	≥10 years (*n* = 51)	*p*-value	≤5yrsVS 6-9 yrs. *p*-value	≤5yrsVS ≥10 yrs. *p*-value	6-9yrsVS ≥10 yrs. *p*-value
Age (yrs)	41.35 ± 9.43	42.60 ± 8.61	47.14 ± 7.13	0.002*	0.740	0.002*	0.021*
ABI	0.93 ± 0.07	0.94 ± 0.07	0.89 ± 0.09	0.012*	0.715	0.039*	0.011*
BMI (Kg/m^2^)	25.46 ± 5.76	25.37 ± 5.61	25.45 ± 5.12	0.996	0.996	1.000	0.997
WC (cm)	88.98 ± 11.67	90.82 ± 11.76	90.55 ± 11.29	0.695	0.709	0.777	0.992
WHR	0.87 ± 0.06	0.88 ± 0.05	0.87 ± 0.037	0.505	0.488	0.929	0.711
SBP (mmHg)	120 ± 21	118 ± 18	127 ± 22	0.054	0.833	0.189	0.053
DBP (mmHg)	82 ± 8	80 ± 7.27	82 ± 8	0.128	0.176	0.189	0.191
Nadir CD4^+^ T cell (cells/mm^3^)	210 ± 224	272 ± 210	304 ± 247	0.122	0.382	0.106	0.754
Current CD4^+^ T cell (cells/mm^3^)	352 ± 220	560 ± 265	596 ± 247	0.001*	0.001*	0.001*	0.745
FPG (mmol/L)	4.40 ± 0.52	4.61 ± 1.16	4.53 ± 0.54	0.413	0.383	0.709	0.850
TC (mmol/L)	4.43 ± 1.12	4.84 ± 1.02	4.57 ± 0.74	0.112	0.101	0.774	0.343
TG (mmol/L)	2.17 ± 0.15	2.22 ± 0.14	2.19 ± 0.14	0.141	0.127	0.397	0.387
HDL-C (mmol/L)	1.19 ± 0.07	1.20 ± 0.06	1.19 ± 0.06	0.582	0.884	0.841	0.552
LDL-C (mmol/L)	2.25 ± 1.07	2.62 ± 0.99	2.38 ± 0.72	0.141	0.125	0.764	0.409

Data are presented as mean ± standard deviation. * = significant at *P* < 0.05. *PAD* Peripheral artery disease, *ABI* Ankle brachial index, *SBP* Systolic blood pressure, *DBP* Diastolic blood pressure, *BMI* Body mass index, *WC* Waist circumference, *WHR* Waist to hip ratio, *CVD* Cardiovascular disease, *TC* Total cholesterol, *TG* Triglyceride, *HDL-C* High density lipoprotein cholesterol, *LDL-C* Low density lipoprotein cholesterol, *FPG* Fasting plasma glucose

**Table 4:** Statistical comparison of traditional cardiovascular risk markers and clinical characteristics based on ART drug regime in thestudy participants using one-way ANOVA

Parameter	AZT/3TC/NVP (*n* = 93)	TDF/3TC/EFV (*n* = 46)	TDF/3TC/ LPV/r (*n* = 11)	*p*-value	AZT/3TC/NVP Vs TDF/3TC/EFV p-value	AZT/3TC/NVP Vs TDF/3TC/LPV/r p-value	TDF/3TC/EFV Vs TDF/3TC/LPV/r p-value
BMI (Kg/m^2^)	25.71 ± 5.54	25.19 ± 5.18	24.04 ± 6.15	0.599	0.865	0.606	0.803
WC (cm)	90.30 ± 11.17	90.09 ± 11.49	88.82 ± 15.29	0.922	0.994	0.915	0.943
WHR	0.88 ± 0.04	0.87 ± 0.05	0.85 ± 0.06	0.041*	0.530	0.039*	0.216
SBP (mmHg)	123 ± 20	122 ± 25	112 ± 11	0.270	0.994	0.2.43	0.306
DBP (mmHg)	82 ± 8	81 ± 7	76 ± 5	0.073	0.823	0.059	0.152
ABI	0.91 ± 0.09	0.94 ± 0.06	0.92 ± 0.09	0.041*	0.039*	0.873	0.671
Nadir CD4+ T cells (cells/mm^3^)	295 ± 250	211 ± 177	216 ± 211	0.100	0.106	0.998	0.998
Current CD4+ T cells (cells/mm^3^)	549 ± 272	443 ± 245	393 ± 237	0.029*	0.041*	0.149	0.834
FPG (mmol/L)	4.40 ± 0.56	4.79 ± 1.13	4.32 ± 0.54	0.019*	0.020*	0.938	0.179
TC (mmol/L)	4.68 ± 1.04	4.46 ± 0.90	4.66 ± 0.60	0.432	0.404	0.997	0.810
TG (mmol/L)	2.20 ± 0.15	2.19 ± 0.14	2.23 ± 0.09	0.671	0.919	0.754	0.647
HDL-C (mmol/L)	1.20 ± 0.07	1.19 ± 0.06	1.18 ± 0.08	0.538	0.611	0.730	0.976
LDL-C (mmol/L)	2.49 ± 1.01	2.28 ± 0.09	2.46 ± 0.54	0.460	0.432	0.997	0.823

Data are presented as mean ± standard deviation. * = significant at *P* < 0.05. *PAD* Peripheral artery disease, *ABI* Ankle brachial index, *SBP* Systolic blood pressure, *DBP* Diastolic blood pressure, *BMI* Body mass index, *WC* Waist circumference, *WHR* Waist to hip ratio, *CVD* Cardiovascular disease, *TC* Total cholesterol, *TG* Triglyceride, *HDL-C* High density lipoprotein cholesterol, *LDL-C* Low density lipoprotein cholesterol, *FPG* Fasting plasma glucose

### Effect of ABI category on CD4^+^ T cell count and cardiovascular risk factors in the study group

The study group stratified into 3 groups (normal ABI = 1–1.3, borderline ABI = 0.9–0.99, low ABI = < 0.9) based on ankle brachial index measurement (Table 5). Total cholesterol and LDL-C significantly varied among the 3 group (*P* < 0.05). Total cholesterol and LDL-C were significantly higher in participants with low ABI compared to those with borderline ABI. LDL-C was significantly higher in study participants with low ABI compared to those who demonstrated normal ABI (Table 5).

**Table 5:** Statistical analysis of anthropometric parameters, clinical characteristics, lipid profile, fasting plasma glucose based on ankle brachial index (ABI) measurement in the study participants

Parameter	Normal ABI (1–1.3) (*n* = 42)	Borderline ABI (0.9–0.99) (*n* = 86)	PAD (> 0.9) (*n* = 22)	*P*-value	Normal ABI VSborderline ABI *P*-value	Borderline ABI VS PAD *P*-value	Normal ABI VS PAD *P*-value
Age (yrs)	44.67 ± 7.85	43.43 ± 9.50	43.14 ± 7.32	0.713	0.735	0.989	0.786
BMI (Kg/m^2^)	25.43 ± 6.05	25.08 ± 5.31	26.77 ± 4.86	0.435	0.941	0.401	0.618
WC (cm)	89.55 ± 12.10	89.28 ± 11.38	94.55 ± 10.39	0.149	0.991	0.135	0.991
WHR	0.87 ± 0.05	0.87 ± 0.05	0.89 ± 0.04	0.468	1.000	0.455	0.532
Nadir CD4	281 ± 230	258 ± 242	250 ± 176	0.833	0.853	0.990	0.853
Current CD4	495 ± 248	507 ± 277	518 ± 267	0.946	0.970	0.985	0.945
Duration on ART (yrs)	6.88 ± 3.14	7.55 ± 3.45	8.32 ± 3.75	0.479	0.555	0.611	0.248
FPG (mmHg)	4.57 ± 1.20	4.49 ± 0.56	4.50 ± 0.62	0.268	0.848	0.998	0.938
TC (mmol/L)	4.58 ± 1.02	4.50 ± 0.99	5.15 ± 0.62	0.019*	0.893	0.014*	0.066
TG (mmol/L)	2.21 ± 0.16	2.19 ± 0.15	2.23 ± 0.08	0.397	0.766	0.390	0.768
HDL-C (mmol/L)	1.20 ± 0.06	1.19 ± 0.07	1.19 ± 0.06	0.942	0.948	0.942	1.000
LDL-C (mmol/L)	2.38 ± 1.00	2.31 ± 0.95	2.94 ± 0.64	0.017*	0.907	0.013*	0.038*

Data are presented as mean ± standard deviation. * = significant at *P* < 0.05. *ABI* Ankle brachial index, *SBP* Systolic blood pressure, *DBP* Diastolic blood pressure, *BMI* Body mass index, *WC* Waist circumference, *WHR* Waist to hip ratio, *CVD* Cardiovascular disease, *TC* Total cholesterol, *TG* Triglyceride, *HDL-C* high density lipoprotein cholesterol, *LDL-C* Low density lipoprotein cholesterol, *FPG* Fasting plasma glucose

**Table 6:** Results of logistic regression analysis: association of risk factors with low ankle-brachial index value in the study group

Parameter	OR	95% CI	*P*-value
BMI	1.052	0.971–1.138	0.214
Age	0.993	0.942–1.046	0.703
WC	1.040	1.000–1.082	0.052
WHR	1.126	0.409–3.107	0.818
CD4+ T cell ≥500 cells/mm^3^	1.000	0.999–1.002	0.801
CD4+ T cell < 200 cells/mm^3^	2.635	0.327–21.05	0.364
Duration of HIV	1.060	0.929–1.210	0.385
Duration on ART	1.094	0.952–1.258	0.203
FPG	0.978	0.546–1.753	0.941
TC	1.784	1.142–2.787	0.011*
TG	6.440	0.269–154.26	0.250
HDL-C	0.215	0.000–208.20	0.215
LDL-C	1.824	1.155–2.880	0.010*
Hypertension	2.115	0.460–9.716	0.335
Family history of CVD	0.956	0.197–4.642	0.956
MetS based on IDF	1.051	0.324–3.408	0.935
Protease inhibitor (LPV/r)	0.678	0.134–3.43	0.638
Hypertension	2.115	0.461–9.716	0.335
Diabetes mellitus	0.328	0.056–1.910	0.215
Viral load ≥20 copies/ml	1.375	0.532–3.55	0.511

* = significant at *P* < 0.05. *CI* Confidence interval, *OR* Odd ratio, *TC* Total cholesterol, *TG* Triglyceride, *BMI* Body mass index, *FPG* Fasting plasma glucose, *WC* Waist circumference, *WHR* Waist hip ratio, *HDL* High-density lipoprotein *LDL* Low-density lipoprotein, *LPV/r* Lopinavir/ritonavir

### Association of low ankle brachial index with cardiovascular risk factors and clinical characteristics in the study group

Low ankle brachial index showed significant association with total cholesterol (OR 1.784, *P* = 0.011) and low-density lipoprotein cholesterol (OR 1.824, *P* = 0.010). TG (OR 6.440, *P* = 0.250), CD4 T cell < 200 copies/ml (OR 2.635, *P* = 0.250), WHR (OR 928.39, *P* = 0.250), hypertension (0R 2.115, *P* = 0.335), detectable viraemia ≥20 copies/ml (OR 1.375, *P* = 0.511) demonstrated high odd ratio for predicting low ABI in the study group (Table 6).

### Correlation analysis between duration on ART and ankle brachial index, triglyceride and viral load in the study group

**Fig. 1:**
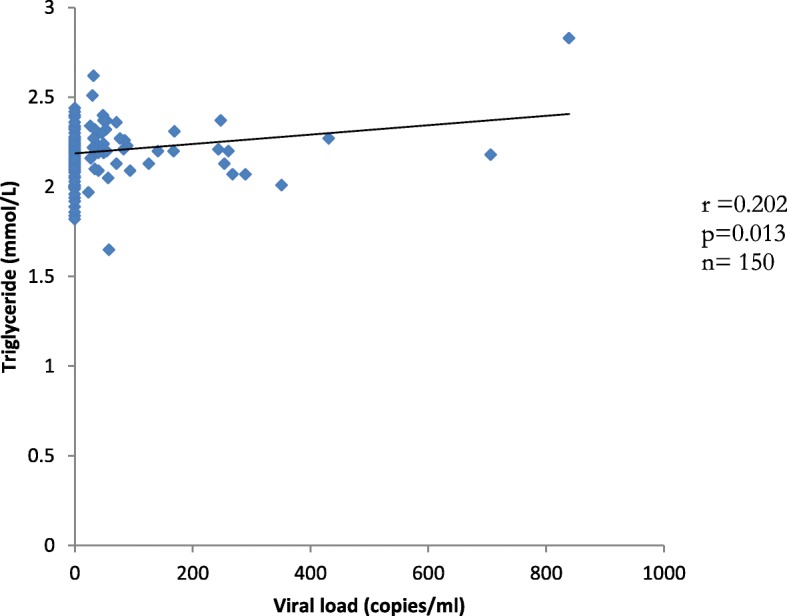
Correlation plot of triglyceride against viral load in HIV-infected study group. The preliminary analysis of the data showed that a significantly positive correlation was observed between triglyceride and viral RNA count

**Fig. 2:**
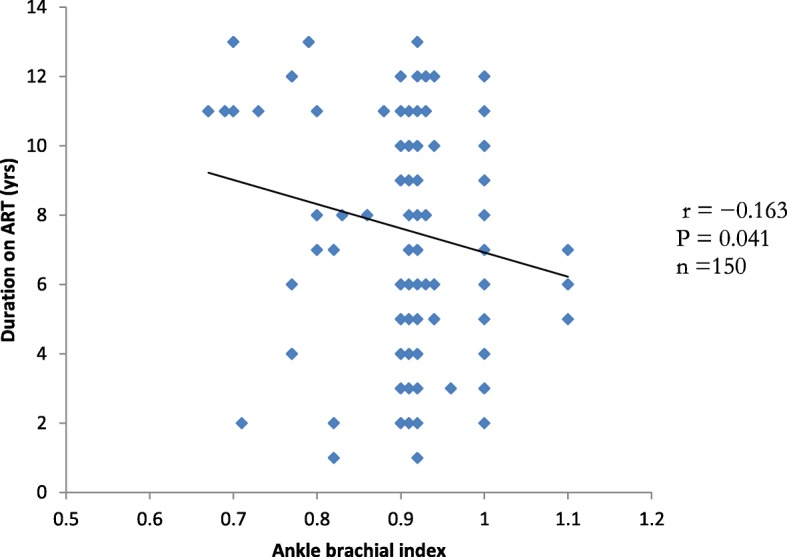
Correlation plot of Duration on ART against ankle brachial index (ABI) in HIV-infected study group. The preliminary analysis of the data showed that a significantly negative correlation was observed between duration of ART (years) and ankle brachial index in the study group

Figure 1 shows a significant positive correlation (*r* = 0.202, *p* = 0.013) between triglyceride and viral load. Figure 2 shows a significant negative correlation (*r* = − 0.163, *P* = 0.041) between duration on ART and ankle brachial index (ABI). This represents an inverse relationship between ABI and duration on ART.

## Discussion

Effectively treated HIV-infected patients still have an elevated risk for cardiovascular morbidity and mortality, which is believed to be associated not only to traditional risk factors, but also to dyslipidemia initiated by HIV and/or antiretroviral treatment [24]. In the general population, carotid intima-media thickness (IMT) evaluated using ultrasonography has been demonstrated to correlate with coronary atherosclerosis [32], and directly associated in older patients with an elevated risk of myocardial infarction and stroke in those without a history of cardiovascular disease [33]. Carotid IMT measurement, notwithstanding, is costly and not easily accessible, instead a noninvasive technique used is the ankle–brachial index (ABI), a simple inexpensive diagnostic test that is a strong indicator of systemic atherosclerosis and peripheral vascular disease and a powerful predictor of mortality from cardiovascular events in the general population [34, 35].

This study evaluated the prevalence of peripheral artery disease (PAD) using ankle brachial index measurement (ABI < 0.9) and its associated risk factors in virologically suppressed HIV-infected participants attending their routine out-patient clinic reviews in University of Ilorin Teaching Hospital and Sabo Oke Medical center Ilorin, Kwara state, Nigeria. The study population was relatively young with a mean age of 43.73 ± 8.74 years and mainly composed of female participants (72.7%). The prevalence of metabolic syndrome, as recorded in study population group using the IDF criteria, is 17.3% compared to 10% recorded in the control group. These findings are consistent with studies of Katherine et al. [36], who reported a prevalence of 14–18% among HIV-infected patients, a study conducted Pirjo et al. [37], also revealed higher prevalence of metabolic syndrome in HIV-infected individuals compared to the uninfected controls with a prevalence rate of 10–14% among HIV-infected individuals with normal glucose tolerance. Treatment with ART can induce severe metabolic complications including lipodystrophy, dyslipidaemia, and insulin resistance and may contribute to the higher prevalence rate observed in the study group [38].

PAD has been shown to correlate with future incident cardiovascular events particularly ischemic strokes and myocardial infarction [39], and can be evaluated using ABI, a low ABI of < 0.9 is suggestive of PAD. A study conducted by Gutierrez et al. [40], showed a relationship between low ABI and elevated carotid intima media thickness, suggesting that in HIV-infected patients, low ABI may be a significant predictor of subclinical atherosclerosis. In this present study, Ankle brachial index was significantly lower in the study group compared to the HIV-negative control group. The study group recorded higher prevalence rate of low ABI; 14.6% compared to 2% prevalence rate observed in the control group. This is consistent with reports by Periard et al. [41], who reported a high prevalence rate (20.7%) of PAD among HIV patients on ART in a Swiss cohort. Findings from this study are also in line with the works of Beckman et al. [42], who reported a 19% increased risk of PAD among HIV- infected veterans.

Different mechanisms may be linked to the premature development of PAD in HIV-infected population. People living with HIV tend to have elevated prevalence of traditional cardiovascular risk factors compared to the un-infected controls, this was observed in this study as HIV-infected study group demonstrated higher prevalence rate of hypertension 15.9%, metabolic syndrome 17.3%, diabetes mellitus 4% compared to the prevalence rate recorded by the control group; hypertension 3.3%, metabolic syndrome 10 and 2% for diabetes mellitus. These risk factors have been linked to future events of atherosclerosis and coronary heart disease. In a study carried out by Guiterrez et al. [40], results showed that traditional CVD risk factors such as hypertension, dyslipidaemia and current smoking status were linked with increased intima media thickness (IMT) within HIV-infected study group.

Another possible mechanism may be linked to combination antiretroviral therapy–related dyslipidaemia, lipodystrophy, and impaired glucose linked to the development of premature atherosclerosis, as previously demonstrated by Maggi et al. [43], and Depairon et al. [44], especially in those on protease inhibitors (PIs). In contrast to these reports, the use of PI did not show a strong associated with low ABI in the present study group. This discrepancy may be due to the mechanisms by which PIs are atherogenic. PIs are associated with usually reversible dyslipidemia, and its long-term may lead to development of atherosclerosis, whereas a short duration of PI exposure may temporarily modify lipid levels without substantial development of atherosclerosis [44]. A negative correlation was however observed between ABI and the duration on ART in this study, this indicates that there is an association between ART and PAD, which needs further clarification. Larger studies with larger number of patients will be necessary to confirm the role of ART and to investigate possible associations of other antiviral agents with PAD.

Another mechanism may result from the direct damage caused by HIV on the arterial wall, leading to inflammatory lesions, as recently demonstrated by Maggi et al. [45]. This is represented by the aneurysmal dilation of large arteries seen in HIV-infected children. In HIV-infected children aged 2–9 years who developed HIV through vertical transmission, [46]. The aortic root size was related with left ventricular dilation, decreased CD4+ T-cell count and elevated viral load. Furthermore, cases of 13 HIV-infected children with cerebral artery aneurysms were reported by Dubrovsky et al., [47]. Reports from this study revealed that CD4 < 200 copies/mm^3^ was associated with a two-fold elevation in the risk of PAD while detectable viraemia was associated with a one-fold increase in the risk of PAD. Such an association has previously been reported by Mercie’ et al. [48], but the underlying mechanism is not clear. These clinical outcomes observed in this study have been supported by other studies that used surrogate CVD markers. In the Women’s Interagency HIV Study (WIHS) and Multicenter AIDS Cohort Study (MACS), a current CD4+ T-cell count < 200 cells/mm^3^ was linked to carotid plaque (defined as a carotid IMT 1.5 mm) [49]. In most of the antiretroviral-treated individuals observed in earlier San Francisco–based research, a nadir CD4+ T-cell count < 200 cells/mm3 was also independently related to carotid IMT [50]. The association of PAD with reduced CD4+ T-cell count may also be linked to patient age, as older age is a risk factor of reduced robust increase in CD4+ T-cell counts after initiation of antiretroviral therapy.

Findings from this study revealed that total cholesterol; LDL-C and TG were significantly higher in the HIV- infected study group compared to the uninfected control group. This is in line with the study of Nsonwu et al. [51], who also reported similar findings. Dyslipidaemia in HIV-infected individuals has been attributed to elevated apolipoprotein levels, increased hepatic synthesis of TG and VLDL-C, decreased clearance of TG, acute-phase proteins, and to the effects of viral infection itself. The levels of total cholesterol (TC) and high-density lipoprotein-cholesterol (HDL-C) has been shown to be rather decreased in the early stages of HIV infection [52]. The progressive decrease in CD4+ T cell lymphocyte counts resulting from destruction by HIV has been however demonstrated to induce a decreased clearance of LDL-C particles, a decrease in HDL-C and an increase in the TG levels which may be positively linked to the level of viraemia [53]. ART has also been shown to affect the metabolism of triglyceride-rich lipoproteins [54].

In addition to the above findings, HIV-infected study participants with altered (low) ABI values recorded significantly higher mean values of TC and LDL-C compared to study participants with normal and borderline ABI values and together with TG predicted low ABI in the study group. These findings may be linked to the fact that LDL-C accumulates in the intima of the artery and initiates the vascular inflammatory process [55]. Oxidized LDL-C and turbulent blood flow occurring at vascular branch points can stimulate adhesion molecules on the endothelial surface of the artery [56]; the stimulation of these adhesion molecules causes activated macrophages and T cells to infiltrate the arterial walls [57]. Monocytes differentiate into macrophages, which take up oxidized LDL-C, forming foam cells, which is critical in the final formation of atheroma’s and development to cardiovascular diseases.

Among HIV-infected participants in this study, the levels of HDL-C were significantly lower compared to un-infected control group. These findings are consistent with the report of Nsonwu et al. [51], who reported that lower levels of HDL among HIV-infected individuals on ART compared to uninfected controls. Lower HDL-C levels may result from HIV infection and/or thrombotic activity, a mediator of endothelial injury and thrombogenesis, or both. The activity of CETP (cholesterol ester transfer protein), which transfers cholesterol esters from HDL-C to apolipoprotein-B–containing proteins [58], is elevated in HIV infection, and its activity inversely correlates with serum HDL levels [59]. This may assist in explaining why HDL-C levels are reduced in HIV infection. Although the reason for elevated CETP activity is still uncertain; CETP functions more efficiently when there is a increased TG level [60], and may aid in explaining the increased activity in HIV-infected patients.

## Conclusion

Detectable viral load count and long term treatment with antiretroviral therapy are associated with dyslipidemia, with elevated risk of peripheral artery disease. Prevalence of PAD in virologically-suppressed HIV-infected participants did not differ from the HIV-negative controls in the population studied.

### Limitations of the study

Our present study is limited by its small sample size and larger studies are required to better characterize risk factors for PAD and its prognosis in this population. There was also no possibility of comparing our results with others in the Nigerian general population as we did not find them in the literature. Accordingly, in the discussion of our results, we compared them with results of the most important worldwide research concerning the prevalence of PAD in the general population. Lastly, this was a single center study in a tertiary hospital; therefore our findings cannot be generalized.

## Additional file

Additional file 1: Questionnaire̓. (DOCX 15 kb)

## Data Availability

The datasets used and/or analyzed during the current study are available from the corresponding author on reasonable request.

## Abbreviations

ABI: Ankle brachial index
AIDS: Acquired Immune Deficiency Syndrome
ART: Antiretroviral therapy
cIMT: Carotid Intima media thickness
CVD: Cardiovascular disease
ELISA: Enzyme-Linked Immunosorbent Assay
HDL-C: high-density lipoprotein cholesterol
HIV: Human Immunodeficiency Virus
LDL-C: Low-density lipoprotein cholesterol
MACS: Multicenter AIDS Cohort Study
NCDs: Non Communicable Diseases
PAD: Peripheral Artery Disease
PLHIV: People Living with Human Immunodeficiency Virus
sICAM-1: Soluble intercellular adhesion molecule-1
TG: Triglyceride
WIHS: Women’s Inter-agency HIV Study
