# Supplementary material for: Prevalence and associated risk factors of peripheral artery disease in virologically suppressed HIV-infected individuals on antiretroviral therapy in Kwara state, Nigeria: a cross sectional study
Source: BMC Public Health. 2019 Aug 20;19:1143. doi: 10.1186/s12889-019-7496-4 (PMC6700806; doi:10.1186/s12889-019-7496-4)
Supplement: Supplementary file 1 — Questionnaire̓. (DOCX 15 kb) [file 12889_2019_7496_MOESM1_ESM.docx]

**APPENDIX 1**

**Questionnaire**

**Prevalence and associated risk factors of peripheral artery disease in virologically suppressed HIV-infected individuals on antiretroviral therapy in Kwara State, Nigeria: a cross sectional study.**

1. Serial Number: ……………………………………………………………….
2. Hospital no…………………………………………………………
3. Gender Male [ ] Female [ ] pregnant [ ] breastfeeding [ ]
4. Age [ ]
5. Ethnicity Yoruba [ ] Nupe [ ] Ibo [ ] Hausa [ ]
6. Weight in Kg ………………………………………………………….
7. Height in meters …………………………………………………………..
8. BMI …………………………………………………………
9. Waist circumference ………………………………………
10. Systolic blood pressure ………………………………………
11. Ankle blood pressure …………………………………………………
12. Ankle brachial index (ABI)………………………………………………
13. Marital Status Single [ ] married [ ] divorced [ ] widowed [ ]
14. Education primary [ ] secondary [ ] tertiary [ ] none [ ]
15. Occupation student [ ] Trader [ ] farmer [ ] civil servant [ ] artisan [ ] unemployed [ ]
16. Religion Muslim [ ] Christian [ ] others [ ]
17. Year diagnosed of HIV ………………………………………………..
18. Baseline Viral load count………………………………………………..
19. Baseline CD4 cell count…………………………………………………
20. Current CD4 count ………………………………………………….
21. ART commencement date…………………………………………..
22. Duration of HIV [ ]
23. Duration of ART [ ]
24. ART pharmacy appointment in the last 3 months Regular [ ] missed [ ] inconsistent [ ]
25. ART Drug regime

TDF|3TC| EFV (300/300/600mg) [ ]

AZT | 3TC | NVP (300/150/200mg) [ ]

Lopinavir | Ritonavir (LPV | r) 200/50mg [ ]

Atazanavir | Ritonavir (ATV | r) 300/100mg [ ]

Others…………………………………………………

1. No of sex partners………………………………………………..
2. Are you on any antihypertensive or sugar lowering drug? Yes [ ] no [ ]
3. If Yes, state the name…………………………………………………..
4. HIV co-infection HBV [ ] HCV [ ] Syphilis [ ] TB [ ]

none [ ]

1. Morbidity related risk factors

- Are you Diabetic Yes [ ] No [ ]
- Do you Smoke Yes [ ] No [ ]
- Are you Hypertensive Yes [ ] No [ ]
- Does any member of your family have hypertension Yes [ ] No [ ]
